# Synthesis and crystal structure of *catena*-poly[[tetra-μ-acetato-copper(II)]-μ-6-eth­oxy-*N* ^2^,*N* ^4^-bis­[2-(pyridin-2-yl)eth­yl]-1,3,5-triazine-2,4-di­amine]

**DOI:** 10.1107/S2056989021002164

**Published:** 2021-02-26

**Authors:** Mayokun J. Ayodele, Travis C. Green, W. A. Chathuri V. Warsapperuma, Malcolm D. E. Forbes, Alexis D. Ostrowski

**Affiliations:** a Department of Chemistry, Center for Photochemical Sciences, Bowling Green State University, Bowling Green, Ohio 43403, USA

**Keywords:** crystal structure, intra­molecular hydrogen bonding, dinuclear copper, triazine, acetate

## Abstract

The title compound, [Cu_2_(C_19_H_23_N_7_O)(C_2_H_3_O_2_)_4_]_*n*_, was obtained *via* reaction of copper(II) acetate with the coordinating ligand, 6-eth­oxy-*N* ^2^,*N* ^4^-bis­[2-(pyridin-2-yl)eth­yl]-1,3,5-triazine-2,4-di­amine. The crystallized product adopts the monoclinic *P*2_1_/*c* space group. The metal core exhibits a paddle-wheel structure typical for dicopper tetra­acetate units, with triazine and pyridyl nitro­gen atoms from different ligands coordinating to the two axial positions of the paddle wheel in an asymmetric manner. This forms a coordination polymer with the segments of the polymer created by the *c*-glide of the *P*2_1_/*c* setting of the space group. The resulting chains running along the *c*-axis direction are held together by intra­molecular N—H⋯O hydrogen bonding. These chains are further packed by dispersion forces, producing an extended three-dimensional structure.

## Abstract 2

Green-colored crystals of the title compound [Cu_2_(C_19_H_23_N_7_O)_2_(C_2_H_3_O_2_)_4_]_*n*_ crystallized in the monoclinic *P*2_1_/*c* space group. The dinuclear Cu center is coordinated by both acetate groups and 6-eth­oxy-*N* ^2^,*N* ^4^-bis­[2-(pyridin-2-yl)eth­yl]-1,3,5-triazine-2,4-di­amine ligands.

## Chemical context   

Dinuclear Cu^II^ groups are recognized for their crucial role as active sites in metalloenzymes and are present in many biological systems (Festa & Thiele, 2011 ▸; Solomon *et al.*, 2014 ▸). They often constitute the catalytically active sites involved in the stepwise oxidative conversions of many small mol­ecules (Pham & Waite, 2014 ▸; Chakraborty *et al.*, 2014 ▸). A well-known series of metalloenyzmes containing dinuclear copper active sites is that of the polyphenol oxidases (*e.g*. catechol oxidase) where the catechol is easily oxidized to quinone products (Ravikiran & Mahalakshmi, 2014 ▸). In recent years, there has been an increased effort to carry over this efficient and selective oxidation into biomimetic models of metalloenzymes (Mahadevan *et al.*, 2000 ▸; Panda *et al.*, 2011 ▸; Marion *et al.*, 2012 ▸).

As part of this quest, significant efforts have been made to identify and better understand the specific structural patterns found at these copper-containing active sites. These patterns have often been found to convey functionalities that define a particular enzyme. This has led to a focus on the basic elements of coordination between the ligands and the metal centers. For example, when designing mimics of catechol oxidase, many model catalysts include the same basic structural elements (Koval *et al.*, 2006 ▸). These models often contain multidentate ligands with at least five coordinating heteroatoms branched off a central ring, all coordinating to the copper centers. This coordination motif and its orientation often provide a unique accessibility for substrate approach, similar to that found in a type-3 active site (Koval *et al.*, 2006 ▸).
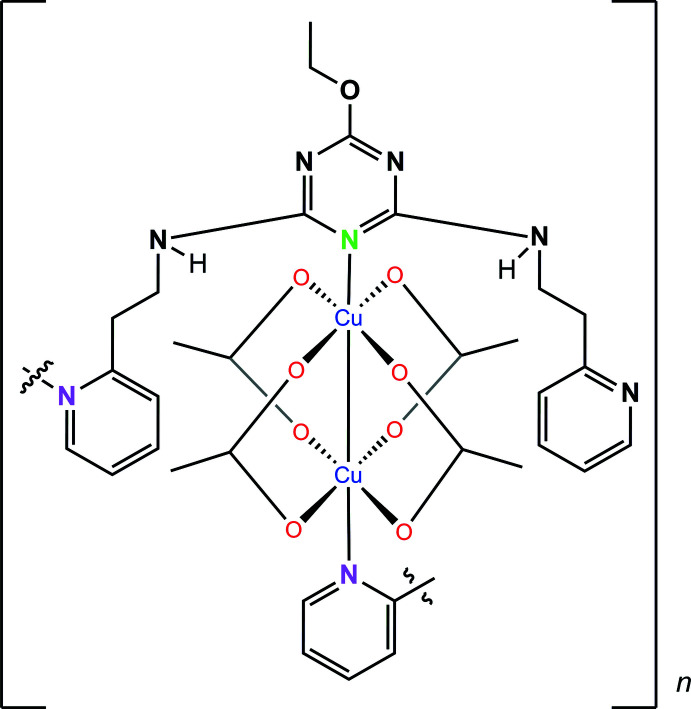


In this paper, we report the crystal structure of a biomimetic complex (**I**) of catechol oxidase synthesized from a multidentate ligand that is coordinated to the copper centers in an unexpected fashion. The complex possesses two nitro­gen coordinating heteroatoms from triazine ligands, which coordinate to the copper centers of the paddle-wheel unit at the axial positions. Additional coordination by the terminal pendant pyridine group on the ligand to another copper paddle-wheel unit creates a continuous coordinated chain linkage.

## Structural commentary   

**Figure 1:**
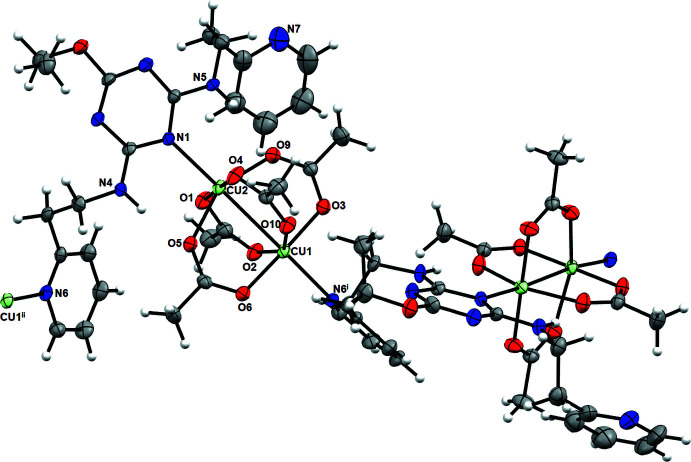
Mol­ecular structure of (**I**) drawn with 50% probability displacement ellipsoids. symmetry code (i) *x*, −*y* + 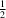, *z* − 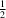; (ii) *x*, −*y* + 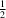, *z* + 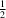. Key: carbon, gray; nitro­gen, blue; copper, light green; oxygen, red.

The title compound (**I**) crystallizes in the space group *P*2_1_/*c*. The mol­ecular structure of (**I**) (Fig. 1 ▸) includes a dinuclear Cu^II^ paddle-wheel unit with both metal ions in slightly Jahn–Teller-distorted octa­hedral environments. The two copper atoms are separated by a Cu1—Cu2 bond distance of 2.7888 (8) Å. As expected in a typical acetate paddle wheel, the acetate groups bridge the Cu atoms in a *μ_2_-O:O′* mode, with the Cu—O bonds lying in the range 1.927 (3)–2.046 (3) Å (Table 1 ▸). The longer Cu—O bonds found for Cu2—O5 [2.046 (3) Å] and Cu2—O9 [2.036 (3) Å] are a consequence of hydrogen-bonding inter­actions involving the O5 and O9 oxygen atoms (see text below for further details). Two triazine ligands coordinate to the copper-acetate paddle-wheel unit in an asymmetric manner, with one Cu atom coordinated to the triazyl nitro­gen, N1, of the central ring on one ligand (green nitro­gen in Scheme 1), and the other Cu coordinated to the terminal pyridyl nitro­gen, N6, of a second ligand (pink nitro­gen in Scheme 1). The two ligands adopt an almost orthogonal orientation to each other. Each of the ligands has their linking alkyl chain adopting a *gauche* geometry, making the two terminal pyridine rings twist away from the central triazine ring.

## Supra­molecular features   

**Figure 2:**
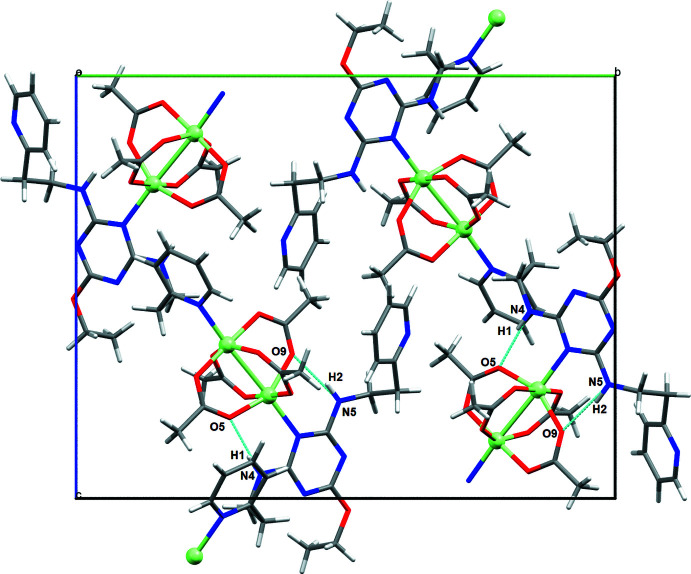
A packing diagram of (I) viewed along the *a* axis showing the one-dimensional network. The N—H⋯·O hydrogen bonds are shown with the dashed light-blue lines. Key: carbon, gray; nitro­gen, blue; copper, light green; oxygen, red.

**Figure 3:**
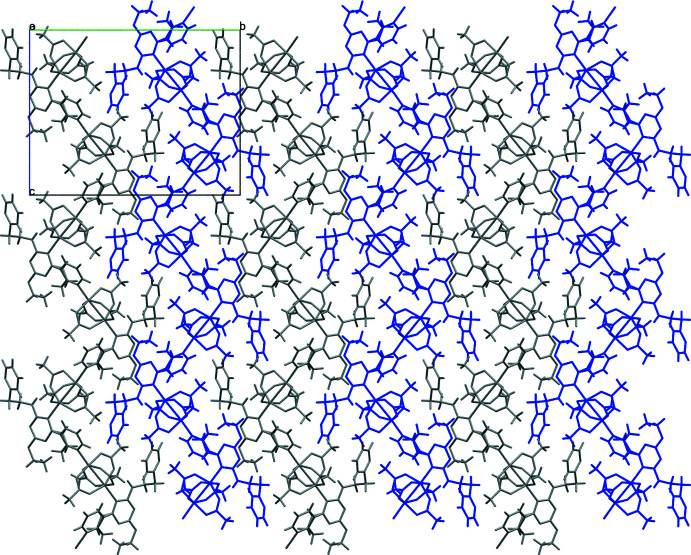
The two-dimensional array of the coordinating networks of (**I**) viewed along the *a* axis.

**Figure 4:**
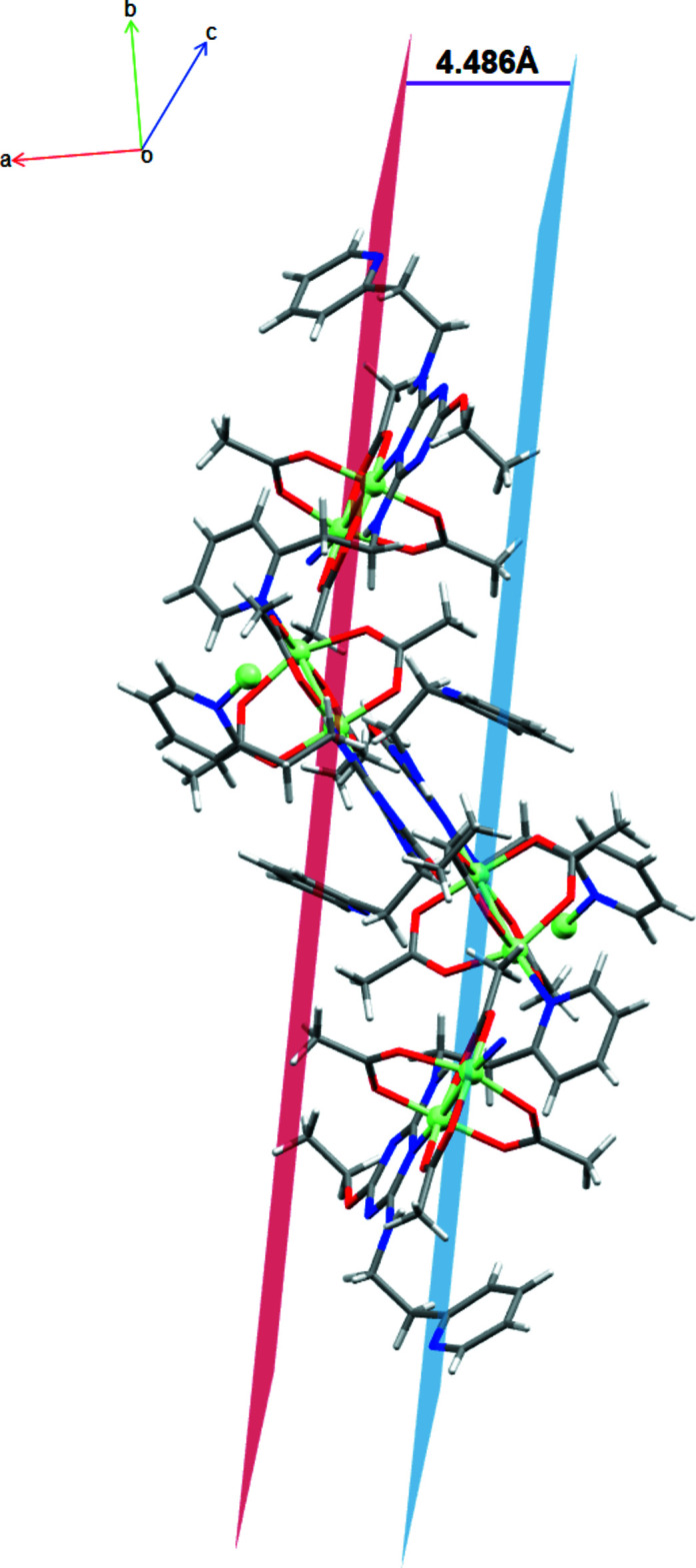
Separated planes of the neighboring one-dimensional networks viewed slightly off the *c* axis. Key: carbon, gray; nitro­gen, blue; copper, light green; oxygen, red.

The copper centers and ligands are linked into a coordination polymer as a consequence of the presence of the *c*-glide in the *P*2_1_/*c* space group. Intra­molecular hydrogen-bonding inter­actions (Table 2 ▸) are observed for only one of the two triazine ligands coordinating to the paddle wheel, as shown in Fig. 2 ▸. These occur between the N4—H1⋯O5 and N5—H2⋯O9 atoms at (H⋯*A*) distances of 1.89 and 1.99 Å, respectively, with the hydrogens on the nitro­gen atoms of the *ortho* branches off the triazine ring pointing towards the oxygen atoms of two of the acetate groups of the paddle wheel. Closely packed arrays of one-dimensional chains, hypothesized to be held together by dispersion forces, form an extended two-dimensional network in the *bc* plane (Fig. 3 ▸), which, with further packing, forms an extended three-dimensional structure. The 1D chains are separated from each other by 4.486 (1) Å, as shown in Fig. 4 ▸.

## Database survey   

A structure survey was carried out on the Cambridge Structural Database (CSD version 5.41, update of August 2020; Groom *et al.*, 2016 ▸). Search results show that although 1,3,5-triazine-2,4-di­amine-derivative complexes with copper, ruthenium and rhodium have been reported (Singh *et al.*, 2010 ▸; Chu *et al.*, 2011 ▸; Massoud *et al.*, 2011 ▸; Chakraborty *et al.*, 2014 ▸), none of these complexes contains a copper(II) acetate [Cu_2_(OAc)_4_ *L*_2_] paddle wheel, as is found in compound (**I**). In all the previously reported structures, each ligand is coord­inated to the metal using at least four of the nitro­gen heteroatoms present. The structure of compound (**I**) presented here is rather different, as each ligand is coordinated to copper through only one nitro­gen heteroatom. In addition, whilst some of the previously reported derivatives contain *ortho*-branched tertiary amines, compound (**I**) contains secondary amines.

## Synthesis, crystallization and catalytic activity   

**Figure 5:**
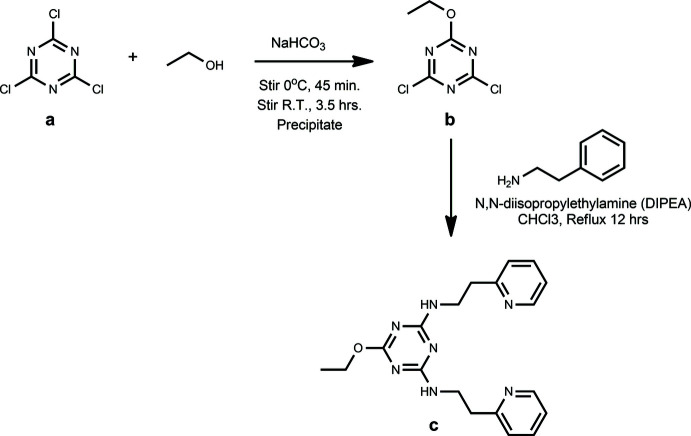
Synthesis of 6-eth­oxy-*N* ^2^,*N* ^4^-bis­(2-(pyridin-2-yl)eth­yl)-1,3,5-triazine-2,4-di­amine.

The triazine ligand (Fig. 5 ▸, *c*) was synthesized by substituting all three chlorines on the cyanuric chloride ring (Fig. 5 ▸, *a*) (Razgoniaev *et al.*, 2016 ▸). The first substitution was completed by chilling 40 mL (0.69 mol) of ethanol in an ice bath. Cyanuric chloride (5.00 g, 27 mmol) and sodium bicarbonate (2.35 g, 28 mmol) were added to the chilled ethanol and stirred in an ice bath for 45 minutes. The reaction mixture was then taken out of the ice bath, stirred at room temperature for 3.5 h and then poured over 20 mL of ice. The resulting precipitate was collected by vacuum filtration. The second and third substitutions were completed by taking the product from step 1 (2.30 g, 12 mmol) (Fig. 5 ▸, *b*) and dissolving it in CHCl_3_. The solution was chilled in an ice bath. 2-(2-Amino­eth­yl)pyridine (3.60 g, 29 mmol) and *N*,*N*-diiso­propyl­ethyl­amine (DIPEA) (3.80 g, 29 mmol) were dissolved in CHCl_3_ and added dropwise to the chilled solution. The reaction was stirred at room temperature for 1 h and stirred at reflux for 12 h. The final product was purified by removing the solvent and taking up the residue in chilled DMF. The product was collected by vacuum filtration and washed at least three times with 15 mL of chilled DMF. The product was obtained as a white powder [yield: 1.75g, 4.8 mmol (40% yield)] and was characterized using ^1^H NMR.

**^1^H NMR (CDCl_3_, 500 MHz)**: δ ppm, 8.6 (*d*, 2H, Ar—H, *a*); 7.7 (*m*, 2H, Ar—H, *b*); 7.2 (*m*, 4H, Ar—H, *c*); 6.6 (*m*, 2H, NH, *d*); 4.5 (*m*, 2H, O—CH_2_, *g*); 4.4 (*m*, 4H, N—-CH_2_–, *e*); 3.1 (*m*, 4H, –N—CH_2_, *f*); 1.4 (*d*, 3H, –CH_3_, *h*)

**Crystal formation of [Cu_2_(C_19_H_23_N_7_O)_2_(C_2_H_3_O_2_)_4_]** ***_n_*** (**I**). The triazine ligand (339.4 mg, 1.0 mmol) was dissolved in chloro­form (20 mL) and a stoichiometric amount of copper(II) acetate (367.3 mg, 1.0 mmol) was dissolved in methanol (20 mL). The two solutions were mixed, and the resulting solution was placed in an ether diffusion chamber for at least four days. Green crystals of (**I**) were filtered off and washed with methanol. The melting point of the crystals was 639–643 K.

**Catalytic activity of [Cu_2_(C_19_H_23_N_7_O)_2_(C_2_H_3_O_2_)_4_]** ***_n_*** (**I**)

**Figure 6:**
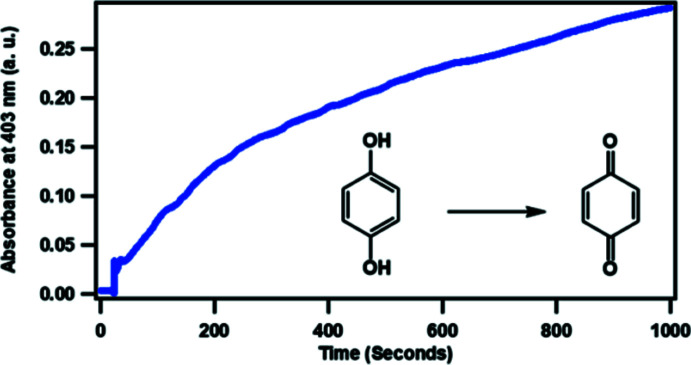
Plot of change in absorbance intensity at 403 nm *vs* time indicating the catalytic oxidation of catechol by (**I**).

The catechol, 1,4-di­hydroxy­benzene, was used to test the catalytic activity of compound (**I**). This catechol is cheap and has good solubility in water. 2 mL of 10^−4^ *M* of compound (**I**) in a chloro­form: methanol (1:1) solution was placed in a cuvette and 10 µL of a 1 *M* solution of the catechol injected. The conversion of the catechol into its quinone derivative (benzo­quinone) was monitored by measuring the absorbance at 403 nm over a period of time. Fig. 6 ▸ shows a continuous increment in absorption at this wavelength, indicating the formation of the product.

## Refinement   

Crystal data, data collection and structure refinement details are summarized in Table 3 ▸. All hydrogen atoms attached to methyl carbons were placed in geometrically calculated positions (C—H = 0.98 Å) and refined using a riding model with displacement parameters [*U*_iso_(H) = 1.5*U*_eq_(C)]. All other carbon–bound hydrogens were placed in geometrically calculated positions (C—H = 0.95–0.99 Å) and were refined as part of a riding model with *U*_iso_(H) = 1.2*U*_eq_(C). Nitro­gen-bound hydrogens were located in a difference-Fourier map and refined using a riding model with fixed displacement parameters [*U*_iso_ (H) = 1.2*U*_eq_(N)], with the N—H bond distance equal to 0.88 Å.

## Supplementary Material

Crystal structure: contains datablock(s) I. DOI: 10.1107/S2056989021002164/cq2041sup1.cif

Structure factors: contains datablock(s) I. DOI: 10.1107/S2056989021002164/cq2041Isup2.hkl

Supporting information file. DOI: 10.1107/S2056989021002164/cq2041Isup3.cdx

CCDC reference: 2064738

Additional supporting information:  crystallographic information; 3D view; checkCIF report

## Figures and Tables

**Table 1:** Selected bond lengths (Å)

Cu1—O6	1.939 (3)	Cu2—O1	1.927 (3)
Cu1—O3	1.946 (3)	Cu2—O4	1.926 (3)
Cu1—O10	2.026 (3)	Cu2—O9	2.035 (3)
Cu1—O2	2.062 (3)	Cu2—O5	2.047 (3)
Cu1—N6i	2.147 (3)	Cu2—N1	2.180 (3)
Cu1—Cu2	2.7889 (8)		

Symmetry codes: (i) *x*, −*y* + {1\over 2}, *z* − {1\over 2}

**Table 2:** Hydrogen-bond geometry (Å, °)

*D*—H⋯*A*	*D*—H	H⋯*A*	*D*⋯*A*	*D*—H⋯*A*
N4—H1⋯O5	0.88	1.89	2.767 (4)	171
N5—H2⋯O9	0.88	1.99	2.857 (4)	168

**Table 3:** Experimental details

Crystal data	
Chemical formula	[Cu_2_(C_19_H_23_N_7_O)(C_2_H_3_O_2_)_4_]
*M*_r_	728.70
Crystal system, space group	Monoclinic, *P*2_1_/*c*
Temperature (K)	100
*a*, *b*, *c* (Å)	8.1495 (8), 21.964 (2), 17.5750 (17)
β (°)	101.457 (4)
*V* (Å^3^)	3083.2 (5)
*Z*	4
Radiation type	Cu *K*α
μ (mm^−1^)	2.25
Crystal size (mm)	0.09 × 0.08 × 0.07

Data collection	
Diffractometer	Bruker AXS D8 Quest CMOS diffractometer
Absorption correction	Multi-scan (*SADABS*; Krause *et al.*, 2015 ▸)
*T*_min_, *T*_max_	0.621, 0.754
No. of measured, independent and observed [*I* > 2σ(*I*)] reflections	32494, 5992, 5118
*R*_int_	0.064
(sin θ/λ)_max_ (Å^−1^)	0.618

Refinement	
*R*[*F* ^2^ > 2σ(*F* ^2^)], *wR*(*F* ^2^), *S*	0.059, 0.128, 1.12
No. of reflections	5992
No. of parameters	411
H-atom treatment	H-atom parameters constrained
Δρ_max_, Δρ_min_ (e Å^−3^)	0.47, −0.58

Computer programs: *APEX2* (Bruker, 2014 ▸), *SAINT* (Bruker, 2013 ▸), *SHELXS97* (Sheldrick, 2008 ▸), *SHELXL2014/7* (Sheldrick, 2015 ▸) *shelXle* (Hübschle *et al.*, 2011 ▸), *Mercury* (Macrae *et al.*, 2020 ▸), *SHELXTL* (Sheldrick, 2008 ▸) and *publCIF* (Westrip, 2010 ▸).

## supplementary crystallographic information

### Crystal data

**Table:**

[Cu_2_(C_19_H_23_N_7_O)(C_2_H_3_O_2_)_4_]	*F*(000) = 1504
*M**_r_* = 728.70	*D*_x_ = 1.570 Mg m^−^^3^
Monoclinic, *P*2_1_/*c*	Cu *K*α radiation, λ = 1.54178 Å
*a* = 8.1495 (8) Å	Cell parameters from 9834 reflections
*b* = 21.964 (2) Å	θ = 3.3–72.4°
*c* = 17.5750 (17) Å	µ = 2.25 mm^−^^1^
β = 101.457 (4)°	*T* = 100 K
*V* = 3083.2 (5) Å^3^	Block, green
*Z* = 4	0.09 × 0.08 × 0.07 mm

### Data collection

**Table:**

Bruker AXS D8 Quest CMOS diffractometer	5118 reflections with *I* > 2σ(*I*)
Radiation source: I-mu-S microsource X-ray tube	*R*_int_ = 0.064
ω and phi scans	θ_max_ = 72.4°, θ_min_ = 3.3°
Absorption correction: multi-scan (SADABS; Krause *et al.*, 2015)	*h* = −9→10
*T*_min_ = 0.621, *T*_max_ = 0.754	*k* = −27→27
32494 measured reflections	*l* = −21→21
5992 independent reflections	

### Refinement

**Table:**

Refinement on *F*^2^	0 restraints
Least-squares matrix: full	Hydrogen site location: inferred from neighbouring sites
*R*[*F*^2^ > 2σ(*F*^2^)] = 0.059	H-atom parameters constrained
*wR*(*F*^2^) = 0.128	*w* = 1/[σ^2^(*F*_o_^2^) + 14.2144*P*] where *P* = (*F*_o_^2^ + 2*F*_c_^2^)/3
*S* = 1.12	(Δ/σ)_max_ < 0.001
5992 reflections	Δρ_max_ = 0.47 e Å^−^^3^
411 parameters	Δρ_min_ = −0.58 e Å^−^^3^

### Special details

**Table:**

Geometry. All esds (except the esd in the dihedral angle between two l.s. planes) are estimated using the full covariance matrix. The cell esds are taken into account individually in the estimation of esds in distances, angles and torsion angles; correlations between esds in cell parameters are only used when they are defined by crystal symmetry. An approximate (isotropic) treatment of cell esds is used for estimating esds involving l.s. planes.

### Fractional atomic coordinates and isotropic or equivalent isotropic displacement parameters (Å^2^)

**Table:**

	*x*	*y*	*z*	*U*_iso_*/*U*_eq_	
Cu1	0.21566 (7)	0.28042 (3)	0.63661 (3)	0.01910 (15)	
N1	0.4958 (4)	0.41259 (15)	0.85305 (17)	0.0176 (6)	
C1	0.5128 (4)	0.39364 (18)	0.9280 (2)	0.0182 (7)	
O1	0.1575 (3)	0.39851 (14)	0.75122 (17)	0.0280 (6)	
Cu2	0.36793 (7)	0.35695 (3)	0.75666 (3)	0.01962 (15)	
N2	0.6024 (4)	0.42400 (16)	0.98968 (18)	0.0221 (7)	
C2	0.5743 (5)	0.46537 (17)	0.8439 (2)	0.0187 (7)	
O3	0.2721 (4)	0.33954 (14)	0.56345 (16)	0.0275 (6)	
N3	0.6693 (4)	0.49737 (16)	0.90069 (19)	0.0238 (7)	
C3	0.6778 (5)	0.47284 (18)	0.9713 (2)	0.0222 (8)	
O4	0.5758 (3)	0.31407 (14)	0.76057 (17)	0.0285 (7)	
N4	0.4395 (4)	0.34253 (15)	0.94256 (18)	0.0214 (7)	
H1	0.3869	0.3209	0.9030	0.026*	
C4	0.7996 (6)	0.4807 (2)	1.1067 (2)	0.0296 (9)	
H4A	0.6922	0.4644	1.1162	0.036*	
H4B	0.8360	0.5137	1.1445	0.036*	
O5	0.2767 (4)	0.28468 (14)	0.80890 (16)	0.0282 (6)	
N5	0.5563 (4)	0.48814 (15)	0.76969 (18)	0.0199 (7)	
H2	0.5024	0.4661	0.7309	0.024*	
C5	0.9278 (6)	0.4313 (2)	1.1184 (3)	0.0359 (11)	
H5A	0.8852	0.3962	1.0859	0.054*	
H5B	0.9517	0.4190	1.1731	0.054*	
H5C	1.0307	0.4462	1.1039	0.054*	
O6	0.1639 (3)	0.22284 (13)	0.71209 (15)	0.0236 (6)	
N6	0.0755 (4)	0.27169 (15)	1.04225 (17)	0.0192 (7)	
C6	0.6206 (5)	0.5454 (2)	0.7528 (3)	0.0292 (9)	
H6A	0.6606	0.5424	0.7033	0.035*	
H6B	0.7179	0.5558	0.7942	0.035*	
C7	0.4917 (6)	0.5963 (2)	0.7464 (3)	0.0347 (10)	
H7A	0.4418	0.5961	0.7933	0.042*	
H7B	0.5484	0.6359	0.7443	0.042*	
N7	0.3853 (6)	0.6155 (2)	0.6104 (2)	0.0441 (10)	
C8	0.3538 (6)	0.5897 (2)	0.6751 (3)	0.0352 (10)	
O9	0.3976 (4)	0.40096 (14)	0.65835 (16)	0.0295 (7)	
C9	0.2070 (7)	0.5581 (3)	0.6758 (3)	0.0451 (12)	
H9	0.1891	0.5392	0.7220	0.054*	
O10	0.4582 (3)	0.25350 (14)	0.66345 (16)	0.0269 (6)	
C10	0.0865 (7)	0.5545 (3)	0.6082 (4)	0.0547 (15)	
H10	−0.0154	0.5334	0.6077	0.066*	
C11	0.1158 (7)	0.5817 (3)	0.5421 (3)	0.0512 (14)	
H11	0.0348	0.5806	0.4951	0.061*	
C19	−0.0735 (5)	0.2493 (2)	1.0075 (2)	0.0266 (9)	
H19	−0.1127	0.2134	1.0283	0.032*	
C18	−0.1729 (5)	0.2754 (2)	0.9435 (2)	0.0298 (9)	
H18	−0.2779	0.2580	0.9207	0.036*	
C17	−0.1163 (5)	0.3274 (2)	0.9132 (2)	0.0289 (9)	
H17	−0.1805	0.3459	0.8680	0.035*	
C16	0.0351 (5)	0.3525 (2)	0.9492 (2)	0.0268 (9)	
H16	0.0747	0.3889	0.9300	0.032*	
C15	0.1289 (5)	0.32336 (17)	1.0141 (2)	0.0189 (8)	
C14	0.2967 (5)	0.34749 (18)	1.0542 (2)	0.0219 (8)	
H14A	0.3140	0.3380	1.1103	0.026*	
H14B	0.2977	0.3923	1.0485	0.026*	
C13	0.4415 (5)	0.32016 (19)	1.0209 (2)	0.0229 (8)	
H13A	0.5495	0.3310	1.0550	0.027*	
H13B	0.4319	0.2752	1.0199	0.027*	
C12	0.2675 (8)	0.6108 (3)	0.5462 (3)	0.0560 (16)	
H12	0.2893	0.6288	0.5000	0.067*	
C20	0.5797 (5)	0.27124 (19)	0.7137 (2)	0.0214 (8)	
C21	0.7455 (5)	0.2386 (2)	0.7185 (3)	0.0311 (10)	
H21A	0.7259	0.1988	0.6933	0.047*	
H21B	0.7992	0.2331	0.7731	0.047*	
H21C	0.8185	0.2630	0.6923	0.047*	
O23	0.7747 (4)	0.50524 (13)	1.02788 (16)	0.0286 (7)	
C23	0.1705 (6)	0.1901 (2)	0.8418 (3)	0.0322 (10)	
H23A	0.0889	0.1602	0.8159	0.048*	
H23B	0.1251	0.2110	0.8823	0.048*	
H23C	0.2746	0.1692	0.8653	0.048*	
C22	0.2058 (5)	0.23605 (18)	0.7830 (2)	0.0217 (8)	
C26	0.3480 (5)	0.38767 (19)	0.5880 (2)	0.0227 (8)	
C25	−0.1298 (5)	0.4153 (2)	0.7018 (3)	0.0346 (10)	
H25A	−0.1744	0.4304	0.6493	0.052*	
H25B	−0.1082	0.4497	0.7379	0.052*	
H25C	−0.2114	0.3878	0.7178	0.052*	
C24	0.0304 (5)	0.38149 (19)	0.7023 (2)	0.0236 (8)	
C27	0.3794 (6)	0.4337 (2)	0.5296 (3)	0.0368 (11)	
H27A	0.3726	0.4138	0.4791	0.055*	
H27B	0.4911	0.4513	0.5466	0.055*	
H27C	0.2950	0.4660	0.5249	0.055*	
O2	0.0277 (3)	0.33809 (13)	0.65513 (16)	0.0266 (6)	

### Atomic displacement parameters (Å^2^)

**Table:**

	*U*^11^	*U*^22^	*U*^33^	*U*^12^	*U*^13^	*U*^23^
Cu1	0.0161 (3)	0.0253 (3)	0.0143 (3)	−0.0009 (2)	−0.0007 (2)	−0.0005 (2)
N1	0.0139 (15)	0.0243 (16)	0.0138 (15)	−0.0021 (12)	0.0008 (12)	−0.0017 (12)
C1	0.0110 (17)	0.0250 (19)	0.0163 (17)	−0.0009 (14)	−0.0030 (14)	−0.0010 (15)
O1	0.0175 (14)	0.0345 (16)	0.0296 (15)	0.0046 (12)	−0.0009 (12)	−0.0067 (13)
Cu2	0.0149 (3)	0.0259 (3)	0.0164 (3)	0.0012 (2)	−0.0010 (2)	−0.0024 (2)
N2	0.0208 (17)	0.0287 (18)	0.0148 (15)	−0.0047 (14)	−0.0014 (13)	−0.0028 (13)
C2	0.0170 (18)	0.0208 (19)	0.0171 (18)	0.0004 (15)	0.0007 (14)	−0.0018 (14)
O3	0.0283 (15)	0.0351 (16)	0.0170 (13)	−0.0067 (13)	−0.0004 (11)	0.0017 (12)
N3	0.0239 (17)	0.0250 (17)	0.0206 (16)	−0.0049 (14)	−0.0004 (14)	−0.0031 (13)
C3	0.0186 (19)	0.026 (2)	0.0202 (19)	−0.0064 (16)	0.0002 (15)	−0.0073 (16)
O4	0.0178 (14)	0.0355 (17)	0.0275 (15)	0.0056 (12)	−0.0067 (12)	−0.0104 (13)
N4	0.0218 (17)	0.0290 (18)	0.0120 (15)	−0.0071 (14)	−0.0002 (12)	0.0005 (13)
C4	0.030 (2)	0.034 (2)	0.021 (2)	−0.0058 (18)	−0.0044 (17)	−0.0054 (17)
O5	0.0323 (16)	0.0338 (16)	0.0166 (13)	−0.0089 (13)	0.0003 (12)	−0.0003 (12)
N5	0.0167 (16)	0.0249 (17)	0.0165 (15)	−0.0057 (13)	−0.0009 (12)	−0.0026 (13)
C5	0.024 (2)	0.053 (3)	0.028 (2)	0.004 (2)	−0.0022 (18)	0.000 (2)
O6	0.0234 (14)	0.0269 (15)	0.0195 (14)	−0.0029 (12)	0.0020 (11)	−0.0010 (11)
N6	0.0144 (15)	0.0258 (17)	0.0152 (15)	0.0028 (13)	−0.0022 (12)	−0.0001 (13)
C6	0.028 (2)	0.032 (2)	0.025 (2)	−0.0065 (18)	−0.0003 (17)	0.0027 (18)
C7	0.039 (3)	0.032 (2)	0.033 (2)	−0.003 (2)	0.007 (2)	0.0005 (19)
N7	0.049 (3)	0.044 (2)	0.036 (2)	0.014 (2)	0.003 (2)	0.0086 (19)
C8	0.041 (3)	0.031 (2)	0.033 (2)	0.008 (2)	0.007 (2)	0.0016 (19)
O9	0.0346 (17)	0.0309 (16)	0.0219 (15)	−0.0077 (13)	0.0028 (12)	0.0004 (12)
C9	0.044 (3)	0.051 (3)	0.040 (3)	0.000 (2)	0.006 (2)	0.000 (2)
O10	0.0175 (14)	0.0353 (16)	0.0261 (15)	0.0030 (12)	−0.0002 (11)	−0.0075 (12)
C10	0.037 (3)	0.068 (4)	0.057 (4)	0.002 (3)	0.002 (3)	−0.003 (3)
C11	0.040 (3)	0.061 (4)	0.044 (3)	0.016 (3)	−0.012 (2)	0.002 (3)
C19	0.021 (2)	0.034 (2)	0.025 (2)	−0.0007 (17)	0.0031 (16)	0.0018 (17)
C18	0.0158 (19)	0.041 (3)	0.029 (2)	0.0032 (18)	−0.0046 (16)	0.0009 (19)
C17	0.026 (2)	0.036 (2)	0.021 (2)	0.0077 (18)	−0.0056 (17)	0.0028 (17)
C16	0.026 (2)	0.029 (2)	0.024 (2)	0.0035 (17)	0.0000 (17)	0.0035 (17)
C15	0.0206 (19)	0.0204 (18)	0.0150 (17)	0.0046 (15)	0.0017 (14)	0.0003 (14)
C14	0.027 (2)	0.0220 (19)	0.0143 (17)	−0.0018 (16)	−0.0007 (15)	0.0003 (14)
C13	0.021 (2)	0.029 (2)	0.0162 (18)	0.0005 (16)	−0.0021 (15)	0.0053 (15)
C12	0.063 (4)	0.065 (4)	0.036 (3)	0.025 (3)	0.000 (3)	0.015 (3)
C20	0.0137 (18)	0.029 (2)	0.0205 (19)	−0.0004 (16)	0.0012 (15)	0.0004 (16)
C21	0.017 (2)	0.037 (2)	0.039 (2)	0.0025 (18)	0.0039 (18)	−0.008 (2)
O23	0.0324 (16)	0.0295 (15)	0.0208 (14)	−0.0093 (13)	−0.0021 (12)	−0.0029 (12)
C23	0.038 (3)	0.034 (2)	0.025 (2)	−0.003 (2)	0.0090 (19)	0.0050 (18)
C22	0.0155 (18)	0.028 (2)	0.0220 (19)	−0.0007 (15)	0.0037 (15)	0.0027 (16)
C26	0.0173 (19)	0.030 (2)	0.0201 (19)	−0.0008 (16)	0.0017 (15)	0.0037 (16)
C25	0.018 (2)	0.038 (3)	0.046 (3)	0.0055 (18)	0.0033 (19)	−0.007 (2)
C24	0.0156 (19)	0.029 (2)	0.026 (2)	0.0016 (16)	0.0032 (16)	0.0020 (17)
C27	0.043 (3)	0.039 (3)	0.028 (2)	−0.003 (2)	0.008 (2)	0.007 (2)
O2	0.0192 (14)	0.0328 (16)	0.0254 (15)	0.0036 (12)	−0.0016 (11)	−0.0059 (12)

### Geometric parameters (Å, º)

**Table:**

Cu1—O6	1.939 (3)	C7—H7B	0.9900
Cu1—O3	1.946 (3)	N7—C12	1.332 (7)
Cu1—O10	2.027 (3)	N7—C8	1.342 (6)
Cu1—O2	2.063 (3)	C8—C9	1.384 (8)
Cu1—N6^i^	2.148 (3)	O9—C26	1.256 (5)
Cu1—Cu2	2.7887 (8)	C9—C10	1.385 (8)
N1—C2	1.349 (5)	C9—H9	0.9500
N1—C1	1.362 (5)	O10—C20	1.250 (5)
C1—N4	1.320 (5)	C10—C11	1.369 (9)
C1—N2	1.356 (5)	C10—H10	0.9500
O1—C24	1.264 (5)	C11—C12	1.380 (9)
Cu2—O1	1.928 (3)	C11—H11	0.9500
Cu2—O4	1.927 (3)	C19—C18	1.374 (6)
Cu2—O9	2.036 (3)	C19—H19	0.9500
Cu2—O5	2.046 (3)	C18—C17	1.379 (6)
Cu2—N1	2.179 (3)	C18—H18	0.9500
N2—C3	1.308 (5)	C17—C16	1.384 (6)
C2—N3	1.336 (5)	C17—H17	0.9500
C2—N5	1.377 (5)	C16—C15	1.397 (5)
O3—C26	1.257 (5)	C16—H16	0.9500
N3—C3	1.343 (5)	C15—C14	1.505 (5)
C3—O23	1.344 (5)	C14—C13	1.539 (6)
O4—C20	1.255 (5)	C14—H14A	0.9900
N4—C13	1.459 (5)	C14—H14B	0.9900
N4—H1	0.8800	C13—H13A	0.9900
C4—O23	1.463 (5)	C13—H13B	0.9900
C4—C5	1.492 (6)	C12—H12	0.9500
C4—H4A	0.9900	C20—C21	1.516 (5)
C4—H4B	0.9900	C21—H21A	0.9800
O5—C22	1.256 (5)	C21—H21B	0.9800
N5—C6	1.416 (5)	C21—H21C	0.9800
N5—H2	0.8800	C23—C22	1.513 (6)
C5—H5A	0.9800	C23—H23A	0.9800
C5—H5B	0.9800	C23—H23B	0.9800
C5—H5C	0.9800	C23—H23C	0.9800
O6—C22	1.259 (5)	C26—C27	1.499 (6)
N6—C19	1.340 (5)	C25—C24	1.500 (6)
N6—C15	1.344 (5)	C25—H25A	0.9800
N6—Cu1^ii^	2.148 (3)	C25—H25B	0.9800
C6—C7	1.522 (6)	C25—H25C	0.9800
C6—H6A	0.9900	C24—O2	1.261 (5)
C6—H6B	0.9900	C27—H27A	0.9800
C7—C8	1.515 (7)	C27—H27B	0.9800
C7—H7A	0.9900	C27—H27C	0.9800
			
O6—Cu1—O3	178.22 (12)	N7—C8—C9	122.0 (5)
O6—Cu1—O10	89.15 (12)	N7—C8—C7	115.2 (5)
O3—Cu1—O10	90.05 (13)	C9—C8—C7	122.8 (4)
O6—Cu1—O2	91.34 (12)	C26—O9—Cu2	130.9 (3)
O3—Cu1—O2	88.55 (13)	C8—C9—C10	119.2 (5)
O10—Cu1—O2	149.63 (11)	C8—C9—H9	120.4
O6—Cu1—N6^i^	91.50 (12)	C10—C9—H9	120.4
O3—Cu1—N6^i^	90.27 (12)	C20—O10—Cu1	132.2 (3)
O10—Cu1—N6^i^	111.64 (12)	C11—C10—C9	119.3 (6)
O2—Cu1—N6^i^	98.70 (12)	C11—C10—H10	120.3
O6—Cu1—Cu2	90.00 (8)	C9—C10—H10	120.3
O3—Cu1—Cu2	88.25 (9)	C10—C11—C12	117.6 (5)
O10—Cu1—Cu2	74.51 (8)	C10—C11—H11	121.2
O2—Cu1—Cu2	75.12 (8)	C12—C11—H11	121.2
N6^i^—Cu1—Cu2	173.68 (9)	N6—C19—C18	123.6 (4)
C2—N1—C1	114.8 (3)	N6—C19—H19	118.2
C2—N1—Cu2	123.6 (2)	C18—C19—H19	118.2
C1—N1—Cu2	121.4 (2)	C19—C18—C17	118.3 (4)
N4—C1—N2	117.1 (3)	C19—C18—H18	120.9
N4—C1—N1	119.0 (3)	C17—C18—H18	120.9
N2—C1—N1	123.9 (3)	C18—C17—C16	119.3 (4)
C24—O1—Cu2	119.6 (3)	C18—C17—H17	120.3
O4—Cu2—O1	178.75 (13)	C16—C17—H17	120.3
O4—Cu2—O9	90.60 (13)	C17—C16—C15	119.0 (4)
O1—Cu2—O9	89.29 (13)	C17—C16—H16	120.5
O4—Cu2—O5	90.00 (13)	C15—C16—H16	120.5
O1—Cu2—O5	89.47 (13)	N6—C15—C16	121.5 (4)
O9—Cu2—O5	149.80 (12)	N6—C15—C14	117.1 (3)
O4—Cu2—N1	88.00 (12)	C16—C15—C14	121.3 (4)
O1—Cu2—N1	93.23 (12)	C15—C14—C13	112.3 (3)
O9—Cu2—N1	105.92 (12)	C15—C14—H14A	109.1
O5—Cu2—N1	104.28 (11)	C13—C14—H14A	109.1
O4—Cu2—Cu1	89.33 (9)	C15—C14—H14B	109.1
O1—Cu2—Cu1	89.44 (9)	C13—C14—H14B	109.1
O9—Cu2—Cu1	75.77 (8)	H14A—C14—H14B	107.9
O5—Cu2—Cu1	74.05 (8)	N4—C13—C14	111.2 (3)
N1—Cu2—Cu1	176.85 (9)	N4—C13—H13A	109.4
C3—N2—C1	114.3 (3)	C14—C13—H13A	109.4
N3—C2—N1	125.5 (3)	N4—C13—H13B	109.4
N3—C2—N5	117.0 (3)	C14—C13—H13B	109.4
N1—C2—N5	117.5 (3)	H13A—C13—H13B	108.0
C26—O3—Cu1	119.9 (3)	N7—C12—C11	124.6 (6)
C2—N3—C3	113.3 (3)	N7—C12—H12	117.7
N2—C3—N3	128.1 (3)	C11—C12—H12	117.7
N2—C3—O23	119.2 (4)	O10—C20—O4	125.2 (4)
N3—C3—O23	112.7 (3)	O10—C20—C21	117.7 (4)
C20—O4—Cu2	118.7 (2)	O4—C20—C21	117.1 (3)
C1—N4—C13	123.3 (3)	C20—C21—H21A	109.5
C1—N4—H1	118.4	C20—C21—H21B	109.5
C13—N4—H1	118.4	H21A—C21—H21B	109.5
O23—C4—C5	111.1 (4)	C20—C21—H21C	109.5
O23—C4—H4A	109.4	H21A—C21—H21C	109.5
C5—C4—H4A	109.4	H21B—C21—H21C	109.5
O23—C4—H4B	109.4	C3—O23—C4	117.1 (3)
C5—C4—H4B	109.4	C22—C23—H23A	109.5
H4A—C4—H4B	108.0	C22—C23—H23B	109.5
C22—O5—Cu2	132.8 (3)	H23A—C23—H23B	109.5
C2—N5—C6	123.2 (3)	C22—C23—H23C	109.5
C2—N5—H2	118.4	H23A—C23—H23C	109.5
C6—N5—H2	118.4	H23B—C23—H23C	109.5
C4—C5—H5A	109.5	O5—C22—O6	124.7 (4)
C4—C5—H5B	109.5	O5—C22—C23	117.2 (4)
H5A—C5—H5B	109.5	O6—C22—C23	118.1 (4)
C4—C5—H5C	109.5	O9—C26—O3	125.0 (4)
H5A—C5—H5C	109.5	O9—C26—C27	116.9 (4)
H5B—C5—H5C	109.5	O3—C26—C27	118.1 (4)
C22—O6—Cu1	118.3 (3)	C24—C25—H25A	109.5
C19—N6—C15	118.2 (3)	C24—C25—H25B	109.5
C19—N6—Cu1^ii^	116.8 (3)	H25A—C25—H25B	109.5
C15—N6—Cu1^ii^	124.9 (2)	C24—C25—H25C	109.5
N5—C6—C7	112.9 (4)	H25A—C25—H25C	109.5
N5—C6—H6A	109.0	H25B—C25—H25C	109.5
C7—C6—H6A	109.0	O2—C24—O1	125.2 (4)
N5—C6—H6B	109.0	O2—C24—C25	117.9 (4)
C7—C6—H6B	109.0	O1—C24—C25	116.9 (4)
H6A—C6—H6B	107.8	C26—C27—H27A	109.5
C8—C7—C6	112.3 (4)	C26—C27—H27B	109.5
C8—C7—H7A	109.2	H27A—C27—H27B	109.5
C6—C7—H7A	109.2	C26—C27—H27C	109.5
C8—C7—H7B	109.2	H27A—C27—H27C	109.5
C6—C7—H7B	109.2	H27B—C27—H27C	109.5
H7A—C7—H7B	107.9	C24—O2—Cu1	130.7 (3)
C12—N7—C8	117.3 (5)		
			
C2—N1—C1—N4	179.7 (3)	C19—C18—C17—C16	−1.8 (7)
Cu2—N1—C1—N4	−5.0 (5)	C18—C17—C16—C15	1.7 (6)
C2—N1—C1—N2	−0.8 (5)	C19—N6—C15—C16	−1.8 (6)
Cu2—N1—C1—N2	174.5 (3)	Cu1^ii^—N6—C15—C16	175.6 (3)
N4—C1—N2—C3	177.2 (4)	C19—N6—C15—C14	−179.9 (3)
N1—C1—N2—C3	−2.2 (6)	Cu1^ii^—N6—C15—C14	−2.6 (5)
C1—N1—C2—N3	3.0 (6)	C17—C16—C15—N6	0.1 (6)
Cu2—N1—C2—N3	−172.2 (3)	C17—C16—C15—C14	178.2 (4)
C1—N1—C2—N5	−177.5 (3)	N6—C15—C14—C13	87.7 (4)
Cu2—N1—C2—N5	7.2 (5)	C16—C15—C14—C13	−90.5 (4)
N1—C2—N3—C3	−1.8 (6)	C1—N4—C13—C14	86.6 (4)
N5—C2—N3—C3	178.8 (3)	C15—C14—C13—N4	69.7 (4)
C1—N2—C3—N3	3.8 (6)	C8—N7—C12—C11	0.4 (9)
C1—N2—C3—O23	−177.8 (3)	C10—C11—C12—N7	−1.7 (9)
C2—N3—C3—N2	−2.0 (6)	Cu1—O10—C20—O4	−1.8 (7)
C2—N3—C3—O23	179.6 (3)	Cu1—O10—C20—C21	178.3 (3)
N2—C1—N4—C13	4.3 (6)	Cu2—O4—C20—O10	0.0 (6)
N1—C1—N4—C13	−176.2 (3)	Cu2—O4—C20—C21	180.0 (3)
N3—C2—N5—C6	−5.8 (6)	N2—C3—O23—C4	4.5 (6)
N1—C2—N5—C6	174.7 (4)	N3—C3—O23—C4	−176.8 (3)
C2—N5—C6—C7	−94.9 (5)	C5—C4—O23—C3	79.3 (5)
N5—C6—C7—C8	−69.1 (5)	Cu2—O5—C22—O6	−5.0 (6)
C12—N7—C8—C9	1.5 (7)	Cu2—O5—C22—C23	174.2 (3)
C12—N7—C8—C7	180.0 (5)	Cu1—O6—C22—O5	1.3 (5)
C6—C7—C8—N7	−87.7 (5)	Cu1—O6—C22—C23	−177.9 (3)
C6—C7—C8—C9	90.8 (6)	Cu2—O9—C26—O3	−4.0 (6)
N7—C8—C9—C10	−2.0 (8)	Cu2—O9—C26—C27	174.7 (3)
C7—C8—C9—C10	179.6 (5)	Cu1—O3—C26—O9	2.4 (6)
C8—C9—C10—C11	0.6 (9)	Cu1—O3—C26—C27	−176.2 (3)
C9—C10—C11—C12	1.1 (9)	Cu2—O1—C24—O2	0.3 (6)
C15—N6—C19—C18	1.7 (6)	Cu2—O1—C24—C25	179.3 (3)
Cu1^ii^—N6—C19—C18	−175.9 (3)	O1—C24—O2—Cu1	−0.9 (6)
N6—C19—C18—C17	0.1 (7)	C25—C24—O2—Cu1	−179.9 (3)

Symmetry codes: (i) *x*, −*y*+1/2, *z*−1/2; (ii) *x*, −*y*+1/2, *z*+1/2.

### Hydrogen-bond geometry (Å, º)

**Table:**

*D*—H···*A*	*D*—H	H···*A*	*D*···*A*	*D*—H···*A*
N4—H1···O5	0.88	1.89	2.767 (4)	171
N5—H2···O9	0.88	1.99	2.857 (4)	168
